# 2021/22 Rugby Europe Injury Surveillance Report: SuperCup, Under-20, and Under-18 Championship

**DOI:** 10.3390/ijerph20031800

**Published:** 2023-01-18

**Authors:** António Miguel Cruz-Ferreira, Agathe Montocchio, Elena Usova-Akula, Philippe Tuccelli, Florent Marty

**Affiliations:** 1 Faculty of Medicine, University of Coimbra, 3000-370 Coimbra, Portugal; 2 CEISUC–Center for Health Studies and Research, University of Coimbra, 3004-512 Coimbra, Portugal; 3 Rugby Europe, 75008 Paris, France

**Keywords:** rugby, epidemiology, athletic injuries, injury surveillance

## Abstract

We conducted a prospective cohort study to determine the incidence rate and characterization of the injuries sustained by players during the 2021/22 season of the Rugby Europe SuperCup, Under-20, and Under-18 championships. Team medics reported the injuries, using an online platform. Ethical approval and informed consent were obtained. The overall incidence of injuries ranged from 33.33 (95% CI: 18.97–54.60) in the under-18s to 83.33 (95% CI: 60.34–112.40) in the under-20s, while in the SuperCup it was 41.35 (95% CI: 30.30–55.18) injuries per 1000 player-match-hours. Injury severity (mean days) was higher in the SuperCup (38.33) and lower in the Under-18 tournament (28.50). Lower limb and soft tissue were the most common type of injuries. Tackles caused almost two-thirds of all injuries. Concussion accounted for 10.0% to 25.6% of all injuries. Our data are consistent with previous reports for similar levels of competition and age grades. The injury incidence was higher in the senior competitions (Under-20s and SuperCup). However, for the under-20s, injury rates were higher than in the SuperCup. This might be related to the competition format; however, more studies need to be conducted in the future. Concussion is a common injury and the protocols used at this level seems to be effective to identify it.

## 1. Introduction

Rugby union is one of the fastest growing sports in the world, played by close to 10 million players across the globe, in all age grades [1]. Being a contact sport, the injury incidence in rugby union is one of the highest among collective sports [2], with several injury surveillance studies published in past years [2,3,4,5,6].

Rugby Europe is the sport’s regional governing body and organizes sevens and fifteens international tournaments and competitions at senior, but also at under-18 and under-20 level. In 2021, despite the COVID-19 pandemic, Rugby Europe successfully launched the SuperCup, a semi-professional league for teams and franchises from all over Europe to compete at the highest level. Clubs and franchises from seven countries participated in the opening season (Portugal, Spain, the Netherlands, Belgium, Israel, Georgia, and Russia) that lasted from September 2021 to May 2022. Each team played six pool games and then two semi-finals and a final were played to find the winner.

In the same year, following the cancelation of all tournaments in 2020, Rugby Europe was able to organize the Under-18 and Under-20 Championships. Eight countries participated in the Under-18 (Georgia, Portugal, Russia, Spain, Romania, Belgium, Netherlands, Czechia) and Under-20 tournament (Portugal, Russia, Germany, Spain, Romania, Belgium, Netherlands, Czechia). Both competitions were played in a play-off format (quarter-finals, semi-finals and finals) during seven days (in October and November, respectively), with just 72 h between each match. Under-18 competitions are the first international experience for athletes in Europe, while the under-20 tournaments are a highly competitive environment with the winner granted a ticket to take part in the World Rugby Under-20 Trophy, the worldwide competition that leads to the World Cup.

Williams S. et al. report that the overall incidence of injuries in senior men’s professional rugby union matches was 81 per 1000 player-match-hours [2]. These numbers are slightly higher compared to those reported for World Rugby Under-20 competitions, where the injury incidence was 57.2 per 1000 player-match-hours. For under-18s, however, the data for international competitions are not available [7].

As part of the new policies and strategic plans of Rugby Europe, an injury surveillance study was implemented for the three events. The World Rugby protocol ‘recognise and remove’ was used to manage concussion in these tournaments.

As sparse data are available regarding European semi-professional club competitions, as well as under-18 and under-20 European international rugby competitions, the aim of this study was to determine the incidence rate and characterization of the injuries which occurred during the 2021/22 season of these Rugby Europe competitions. These data can be valuable for doctors, researchers, coaches, and athletes involved in the sport, aiming at the prevention of injury burden in sport [8], and to benchmark the injury incidence and severity at this level of the sport.

## 2. Materials and Methods

A prospective cohort study was conducted during the 2021/22 Rugby Europe SuperCup, as well as during the 2021 Under-18 and Under-20 Rugby Europe Championships. The definitions and procedures used were compliant with the international consensus statement on injury surveillance studies for rugby [9], and the methodology was similar to that used in previously published studies. The reporting of this study conforms to the STROBE statement [10].

Informed consent was collected from the participants prior to the beginning of the tournaments, included in the terms of participation. Ethical approval was obtained from the ethical committee of the Portuguese Centro Regional Health Administration and institutional collaboration was granted by Rugby Europe.

All players taking part in the three competitions were included in the study. The data were collected online, using a specific report instrument modeled after the consensus statement on injury definitions and data collection procedures in studies of injuries in rugby union [9]. Team medics would report the injuries after the matches and the researchers would maintain follow up until recovery. In the report form, the details of each injury were collected: date of injury; playing position (forward, back); period of the game (quarter of the first and second half, extra time); contact or no-contact injury; activity at the time of injury (tackling, being tackled, collision, scrum, line-out, ruck, maul, or other), location of the injury (head/neck, upper limb, trunk, or lower limb), type of injury (bone, joint/ligament, muscle/tendon, skin, brain/spine/peripheral nervous system, or other), field location [9]. In the ‘Laws of the Game’ [11], a description of the various types of activities in rugby union is provided. Only time-loss injuries were considered (those leading to absences from training and matches for more than 24 h). The injury severity was defined as the number of days an injured player was out from training and competition.

The match exposures were calculated as follows: 15 players being exposed for 80 min per game per team (70 min for the under-18s). No allowances were made for players removed from the match (yellow cards, red cards, or medical treatment). The rate of injury is reported as injuries per 1000 player match-hours. Injury data are reported proportionally (%). Severity is reported as the mean number of days. Ninety-five percent confidence intervals (95% CIs) were calculated for the injury rate.

The data was analyzed using the software SPSS Statistics for Windows v.22.0.

## 3. Results

During the SuperCup, 26 matches were played (as one match in the pool stages was cancelled due to the Russia–Ukraine conflict) and a total of 43 injuries were reported, corresponding to an overall injury incidence rate of 41.35 (95% CI: 30.30–55.18) injuries per 1000 player-match-hours. The average severity was 38.33 (95% CI: 13.10–63.56) days. Twelve matches were played during the under-18 competition and twelve more at the under-20 event. Fourteen injuries were reported in the first competition, corresponding to an overall incidence rate of 33.33 (95%CI: 18.97–54.60) injuries per 1000 player-match-hours. In the under-20s tournament, 40 injuries were reported, corresponding to an incidence rate of 83.33 (95% CI: 60.34–112.40) injuries per 1000 player-match-hours. The average severity was 32.62 and 28.50 days, respectively.

Lower limb and soft tissue injuries (mainly sprain/ligament injuries) were the most frequently reported. Contact events, especially tackles, were the activities most prone to injury. Concussion accounted for 10.0–25.6% of all injuries reported, being one of the most frequent injuries reported. Table 1, Table 2 and Table 3 summarize the injury data reported during the tournament.

## 4. Discussion

Despite the significant number of studies in adult rugby, at youth level (under-18) reports are sparse [11,12,13], especially those reporting injuries in international players and competitions [14]. Most studies for school and under-18 athletes report injury rates below 47 injuries per 1000 player-match-hours, around half of what is reported for senior adult male international competitions [6]. On average, these players are absent from training and competition for more than 3 weeks (21 days) [5,13,14]. Cruz-Ferreira et al., Sewry et al. and Solis-Mencia et al. published three papers in recent years, with data from under-18 players competing at national [12,13] and international level [14]. The injury incidence rates reported varied significantly from 17 injuries per 1000 player-match-hours in the South African competitions, to 138 injuries per 1000 player match-hours for the Spanish under-18 players competing in international tournaments.

For the senior under-20 competitions, data on injuries is sparse and mainly consists of reports from the World Rugby Under-20 Trophy and Championships. Fuller and Taylor reported that the overall incidence of injury was 49.7 injuries per 1000 player-match-hours in a series of 8-year injury surveillance studies at both events, with a mean severity of 32.2 days [7].

At semi-professional international senior club level, we were not able to find any relevant study to compare our data against. However, considering the meta-analysis published in 2018 by Yeomans at al. [4], regarding the injury incidence at the amateur level in rugby, we know that the match injury incidence rate at this level (46.8 per 1000 player-match-hours) is lower in comparison with professional cohorts, but higher than those reported for academy players, under-18s and school boys [4].

Regarding the location and type of injuries, all studies agree that lower limb and soft tissue injuries are the most frequent, and contact preceded the majority of injuries, with tackles being the events most prone to injuries [5,6,12,13,15]. Concussion is a major concern and accounts for up to a quarter of all injuries [5,6,12,13,15].

Our findings confirmed the high incidence rate of injuries in rugby, even at these levels. As expected, the incidence rates were higher in the senior competitions (SuperCup and Under-20 Championship) and lower in the under-18s event.

We were also able to confirm that most injuries occur after contact events, something that is also in line with all previous studies. Notably, tackles (both tackling and being talked) accounted for close to two-thirds of all injuries reported by the teams. This highlights the urge identified by World Rugby to improve the rules and regulations regarding this essential part of the game, but also the importance of practicing and perfecting the tackle technique, from the players’ and coaches’ side.

Concussion accounted for between 10 and 25.6% of all injuries reported. This makes concussion one of the most frequent injuries in the sport, thus confirming the risks associated and the need to consider all strategies to mitigate it. Among the tools available to handle concussion are the management protocols in place, both the Head Injury Assessment ‘temporary replacement’ and ‘recognise and remove’. These protocols help the medics with the early detection and removal of the player from the field, allowing for the team not to be in numerical inferiority, by even allowing substituted athletes to rejoin the game, in the event of a removal of a player with a concussion (or suspected concussion). This helps to lower the cut-off for pitch removal, in situations where concussion is suspected, while also decreasing the risk of conflict between medics and both staff and players. As recognise and remove is the protocol used for concussion management in these competitions, considering that concussions reported are in the same range of incidences reported for even the elite game [6,7,16], it gives us a clear sign that it is working. It also highlights that our focus should be on the early identification and removal of concussed (or suspected concussed) players, and that it must remain as the priority in the sport.

The injury incidence rates for the Under-20 Championship were higher than those reported for the SuperCup. It is, nevertheless, important to highlight that this is also a senior competition that grants the winner access to the World Rugby Trophy (top 20 teams in the world) with three matches/teams played over just 1 week having a higher incidence rate than the SuperCup. The latter is also a senior competition but is played throughout the year with a minimum of 1 week’s rest between matches. In addition, the structure of the competition at the under-20 level makes all the matches all-or-nothing events, with a defeat leading to elimination from the tournament, whereas at the SuperCup level only the final two matches are played in a play-off style, allowing for recovery from an upset in the pool stage. This inevitably decreases the importance of the result in each event and might have an impact on the approach from players and staff and in terms of the risks taken, which might lead to a higher incidence rate.

Despite the relevant findings from our study, more injury surveillance studies at this level need to be conducted to validate/increase the value of these findings. Furthermore, we believe that these findings should make the organizers and teams involved in these competitions, especially the Under-20s Championship, reconsider the competition schedules, aiming at player welfare.

Finally, as a tender has been announced for a scientific partnership to conduct injury surveillance studies in Rugby Europe’s tournaments, our report can be used as a benchmark for future studies.

## 5. Conclusions

Despite the limitations (reduced number of competitions, and teams; only one season; injuries reported by the team’s medical staff), this study provided benchmark data for injuries in Rugby Europe competitions. The injury incidence rates in Rugby Europe tournaments during the 2021/22 season were within the expected range for non-tier 1 adult rugby and for youth competitions, according to the previous literature. Specific data for these levels of competitions (tier 2 elite international adult and under-18) is sparse and this study will be a benchmark for future comparisons. Notably, the injury burden in these competitions, with an injury or two reported per match, must trigger the implementation of active strategies aiming at its mitigation, including injury prevention programs and the study of alternative competition formats for the under-20 tournament. Concussion is a frequent injury in rugby union, including in Rugby Europe competitions, therefore all agents must remain aware of the problem and aim at the implementation of best practices aiming at its mitigation—education, HIA (Head Injury Assessment) replacement protocol, injury surveillance, and prevention.

## Figures and Tables

**Table 1:** Exposure, injury incidence, and severity in Rugby Europe competitions.

	Teams	Matches	Total Exposure ^1^ (Player-Match-Hours)	Injuries Reported	Injury Incidence Rate (Injuries per 1000 Player-Match-Hours, 95% CI)	Severity (Days)
SuperCup	8	26+	1040	43	41.35 (30.30–55.18)	38.33
U-20 Championship	8	12	480	40	83.33 (60.34–112.40)	32.62
U-18 Championship	8	12	420	14	33.33 (18.97–54.60)	28.50

Legend: 1—player-match-hours (number of players *x* hours played *x* number of matches played); CI—confidence interval; U-18—under 18; U-20—under 20; +—one match from the pool phase was not played due to the beginning of the Russia–Ukraine conflict.

**Table 2:** Injury location, type, and cause in Rugby Europe competitions.

	SuperCup (Senior Men, %)	Under-20 Championship (Senior Men, %)	Under-18 Championship (Boys, %)
**Injury Location**			
Head/face and neck/cervical spine	32.6	15.0	28.6
Upper limb	20.9	22.5	14.3
Lower limb	44.2	52.5	50.0
Trunk/Abdomen	2.3	10.0	7.1
**Side**			
Left	37.2	40.0	42.9
Right	41.9	40.0	35.7
Bilateral/Not applicable	20.9	20.0	21.4
**Type of Injury**			
Concussion	25.6	10.0	21.4
Fracture	7.0	2.5	-
Other bone injury	2.3	2.5	-
Dislocation/subluxation	4.7	12.5	7.1
Sprain/ligament injury	25.6	35.0	28.6
Muscle rupture/strain/tear/cramps	14.0	7.5	7.1
Tendon injury/rupture/tendinopathy	2.3	2.5	-
Haematoma/contusion/bruise	9.3	25.0	14.3
Dental injury	-	-	7.1
Laceration	9.3	-	-
Other	-	2.5	14.3
**Was the Injury Caused by Contact?**			
Yes	83.7	92.5	85.7
No	16.3	7.5	14.3
**Type of Contact (Percentages Related to Contact Injuries Only)**			
Tackle	69.4	57.9	66.6
Being tackled	33.3	31.6	33.3
Tackling	36.1	26.3	33.3
Ruck	8.3	13.2	8.3
Maul	8.3	2.6	8.3
Scrum	-	7.9	-
Lineout	2.8	-	-
Open game collision	2.8	15.8	16.7
Other	8.3	2.6	-

**Table 3:** Player’s position, round of the competition, field location, and time of injury in Rugby Europe competitions.

	SuperCup (Senior Men, %)	Under-20 Championship (Senior Men, %)	Under-18 Championship (Boys, %)
**Player Position**			
Forward	53.5	52.5	50.0
Back	46.5	47.5	50.0
**Round**			
Round 1	13.9	45.0	28.6
Round 2	23.3	25.0	42.8
Round 3	16.2	30.0	28.6
Round 4	23.3	-	-
Round 5	4.7	-	-
Round 6	-	-	-
Semi-final	13.9	-	-
Final	4.7	-	-
**Field Location**			
Defensive 22 m	30.2	17.5	21.4
Between the defensive 22 m and midfield	39.5	22.5	14.3
Between midfield and the offensive 22 m	16.3	40.0	64.3
Offensive 22 m	14.0	10.0	-
Not applicable	-	10.0	-
**Match Period**			
First 20 min	20.9	20.0	21.4
Last 20 min of the first half *	32.6	7.5	35.8
First 20 min of the second half	23.3	27.5	21.4
Last 20 min of the second half *	23.3	40.0	21.4
Not applicable	-	5.0	-

Legend: * for the Under-18 competition we must read the last 15 min of the first and second halves.

## Data Availability

Data are available upon reasonable request.
